# Downregulation of L1CAM inhibits proliferation, invasion and arrests cell cycle progression in pancreatic cancer cells *in vitro*

**DOI:** 10.3892/etm.2014.1519

**Published:** 2014-02-05

**Authors:** QIWEN BEN, WEI AN, JIAN FEI, MAOJIN XU, GUIXIANG LI, ZHAOSHEN LI, YAOZONG YUAN

**Affiliations:** 1 Department of Gastroenterology, Ruijin Hospital, Shanghai Jiaotong University, Shanghai, P.R. China; 2 Department of Gastroenterology, Changhai Hospital of Second Military Medical University, Shanghai, P.R. China; 3 Department of General Surgery, Ruijin Hospital, Shanghai Jiaotong University, Shanghai, P.R. China; 4 Department of Endocrinology, Changhai Hospital of Second Military Medical University, Shanghai, P.R. China

**Keywords:** L1CAM, pancreatic cancer, proliferation, invasion, cell cycle

## Abstract

The aim of the present study was to establish the effect of silencing L1 cell adhesion molecule (L1CAM) on the proliferation, invasion, cell cycle progression and apoptosis of pancreatic cancer cells, and to determine the potential molecular mechanisms that are involved. The human Capan-2 pancreatic cancer cell line was infected with lentivirus-mediated short hairpin RNA (shRNA) to target L1CAM. Cell proliferation and invasion were analyzed using cell counting kit-8 and Transwell assays, respectively, and cell cycle progression and apoptosis were analyzed using flow cytometry. L1CAM protein expression in Capan-2 cells decreased following shRNA-L1CAM infection. Furthermore, knockdown of L1CAM significantly inhibited cell proliferation and reduced the number of invasive cells, while increasing the percentage of cells in the G0/G1 phase (P<0.05). However, the effect on apoptosis was not identified to be statistically significant. In addition, L1CAM silencing may induce activation of p38/extracellular signal regulated kinase 1/2. Downregulation of L1CAM may inhibit proliferation, invasion and arrests cell cycle progression in pancreatic cancer via p38/ERK1/2 signal pathway, and therefore, L1CAM may serve as a potential target for gene therapy in pancreatic cancer.

## Introduction

Pancreatic cancer is ranked as the fourth leading cause of cancer-related mortalities in the United States (1,2) and the sixth leading cause of mortality in China (3). Pancreatic cancer is characterized by a highly malignant phenotype that is associated with early metastasis and chemoresistance. Although resection of the tumor is considered the primary option for a successful cure (4), the five-year survival rates remain at <5%. Therefore, understanding the underlying molecular mechanisms of invasion and metastasis in pancreatic cancer is central to identifying effective therapeutic targets.

L1 cell adhesion molecule (L1CAM), a 200–220 kDa transmembrane glycoprotein, is a member of the neuronal immunoglobulin superfamily of cell adhesion molecules. L1CAM was originally considered to be a stimulator of neurite outgrowth in the peripheral nervous system due to its involvement in establishing central nervous structures. Mutations in the gene that encodes for L1CAM may result in a variety of developmental defects in humans, including corpus callosum hypoplasia, mental retardation and spastic paraplegia (5). In addition, studies have found that L1CAM is aberrantly expressed in a variety of tumor types, including human gliomas, non-small cell lung cancer, and ovarian, colorectal and pancreatic cancer (6–9).

The presence of L1CAM in tumor tissue and cultured cells has been correlated with poor prognosis and advanced-stage pancreatic cancer (10,11). Positive L1CAM expression has been reported in ~80% of analyses of pancreatic tumor samples and cell lines. Furthermore, L1CAM has been associated with metastasis and angiogenesis during tumor progression by promoting cancer cell adhesion to endothelial cell monolayers, and via transendothelial migration (12). A combined treatment with an L1CAM antibody and gemcitabine or paclitaxel in severe combined immunodeficiency mouse models reduced the growth of subcutaneous pancreatic Colo357 tumors more efficiently than treatment with the cytotoxic agent alone (7). These data demonstrated the central role of L1CAM in the tumorigenesis of pancreatic cancer.

In the present study it was hypothesized that the suppression of L1CAM expression in human pancreatic cancer cells may inhibit tumor progression. To investigate this hypothesis, L1CAM was silenced in Capan-2 pancreatic cancer cells and the effect on proliferation, apoptosis, cell cycle progression and invasion was examined. In addition, the potential role of LICAM in the activation of intracellular signaling pathways was investigated.

## Materials and methods

### Cell culture

Human pancreatic cancer cell lines, Capan-2, PANC-1, AsPC-1, BxPC-3, SW-1990, Patu-8988 and CFPAC-1, were purchased from the Cell Bank of Type Culture Collection of Chinese Academy of Sciences (Shanghai, China). Cells were maintained in RPMI-1640 medium (Invitrogen, Carlsbad, CA, USA) supplemented with 10% fetal bovine serum (FBS; Invitrogen), 100 U/ml penicillin and 100 μg/ml streptomycin (Invitrogen). Cells were incubated at 37°C in a humidified atmosphere of 5% CO_2_ and used for the assays during the exponential phase of growth. The study was approved by the Ethics Committee of Ruijin hospital, Shanghai Jiaotong University, Shanghai, China.

### Construction of recombinant lentivirus and cell infection

Small-interfering RNA (siRNA; 5′-AGGGAUGGUGUCCACUUCAAATT-3) was used to downregulate the L1CAM expression and a non-silencing fragment (5′-TTCTCCGAACGTGTCACGT-3′) served as the negative control. The synthesis of siRNA was conducted by Shanghai GenePharma Co., Ltd (Shanghai, China). Short hairpin RNA (shRNA) fragments were hybridized with synthesized sense and antisense oligonucleotides. The hybridized shRNA fragments were cloned into the pGLV-U6-EGFP plasmid to yield pGLV-Sh-L1CAM plasmid. The correct insertions of the shRNA cassettes were confirmed using direct sequencing.

The shRNA-containing plasmid, pGLV-sh-L1CAM, as well as two imperative elements for virus packaging (VSVG and Gag/pol/rev plasmid), were co-transfected into 293T cells with Lipofectamine 2000. After filtering the collected medium through 0.45 μm-filters, the virus was concentrated by centrifugation at 4,000 × g (Eppendorf, Hamburg, Germany) for 15 min followed by 2 min at 1,000 × g. The concentrated virus was stored at −80°C and the titers of the lentiviral vectors were determined via dilution using fluorescence microscopy (IX71; Olympus, Tokyo, Japan).

### Lentivirus infection in Capan-2 cells

Capan-2 cells were plated at a density of 5×10^4^ cells/well in six-well plates and incubated for 24 h at 37°C and 5% CO_2_. A recombinant lentivirus encoding for shRNA against L1CAM in serum-free growth medium was added at a multiplicity of infection of 50 and, after incubation for a further 2 h, the serum-containing growth medium was added to the cells. The reporter gene expression was assessed by fluorescence microscopy 96 h after infection.

### Polymerase chain reaction (PCR)

The cDNA template was synthesized according to the manufacturer’s instructions (Takara, Tokyo, Japan). PCR was performed in a 25-μl reaction mixture containing 2 μl RT products and β-actin served as the internal control. The amplification products were separated by electrophoresis on a 1.5% agarose gel using the following primers: L1CAM sense, 5′-GACTACGAGATCCACTTGTTTAAGGA-3′ and antisense, 5′-CTCACAAAGCCGATGAACCA-3; β-actin sense, 5′-GCTCCTCCTGAGCGCAAG-3′ and antisense, 5′-CATCTGCTGGAAGGTGGACA-3′.

### Western blot analysis

Total protein was isolated from the cells at the exponential growth phase and the protein concentration was measured using a Bio-Rad assay (Hercules CA, USA). The proteins were transferred to polyvinylidene fluoride membranes following SDS-PAGE and probed with their corresponding primary antibodies (LICAM; Abcam, Cambridge, UK) in a blocking buffer (5% non-fat milk) at 4°C. The immunoreactive bands were detected by chemiluminescence (Pierce Biotechnology, Inc., Rockford, IL, USA) according to the manufacturer’s instructions. GAPDH served as the control to verify that there was equal protein loading.

### Proliferation assay

Cell proliferation assays were performed using cell counting kit-8 (CCK-8; Beyotime Biotechnology, Shanghai, China). The Capan-2 cells were seeded in 96-well plates at a density of 5×10^3^ cells/well, and collected at 24, 48, 72, 96, 120 and 144 h after infection. The absorbance was read at 450 nm using a microplate ELISA reader (SpectroMax 190; Molecular Devices, Sunnyvale, CA, USA).

### Detection of apoptosis

Cells were harvested 48 h after infection, washed in phosphate buffered saline (PBS) and resuspended in 0.1 M PBS. A total of 1×10^6^ cells was collected, washed in PBS and resuspended in Annexin V-fluorescein isothiocyanate (Biouniquer Technology Co., Hangzhou, China) for 10 min. The level of apoptosis was determined using an Annexin V/APC kit and propidium iodide (PI; Biouniquer Technology Co.) according to the manufacturer’s instructions. Following the addition of 50 mg/ml PI and 20 mg/ml RNase A, the cells were incubated in the dark for 20 min at 4°C and analyzed by flow cytometry (FACS420; BD Biosciences, San Jose, CA, USA).

### Cell cycle assay

Cells were harvested 48 h after infection, washed and resuspended in 0.1 M PBS. The cells at a concentration of 1.0×10^6^ cells/ml were fixed in 1 ml pre-cooled 70% alcohol overnight at 4°C. Following incubation with 50 mg/ml PI and 20 mg/ml RNase A for 30 min at 4°C in the dark, the cell cycle distribution was analyzed using flow cytometry.

### Cell invasion assay

Cell invasion assays were conducted using an 8.0-μm Millicell-24 cell culture insert plate (Millipore, Bedford, MA, USA). Cells at a concentration of 1.0×10^6^ cells/ml were added to the upper compartment of the transwell insert and 500 μl medium, with 10% FBS as a chemoattractant, was added to the lower chamber and the plates were incubated at 37°C for 24 h. The noninvasive cells and Matrigel were removed by scraping using sterile cotton swabs. The cells that had invaded were stained with hematoxylin (Biouniquer Technology Co.) and counted under a microscope (magnification, ×100; Nikon, Tokyo, Japan). Cells were counted in five randomly selected fields and the assays were performed in triplicate.

### Statistical analysis

Data were analyzed using SPSS 19.0 software (IBM, Armonk, NY, USA) and are expressed as mean ± standard deviation. Differences were determined using Student’s two-tailed t-tests and P<0.05 was considered to indicate a statistically significant difference.

## Results

### L1CAM mRNA expression in pancreatic cancer cell lines

**Figure 1:**
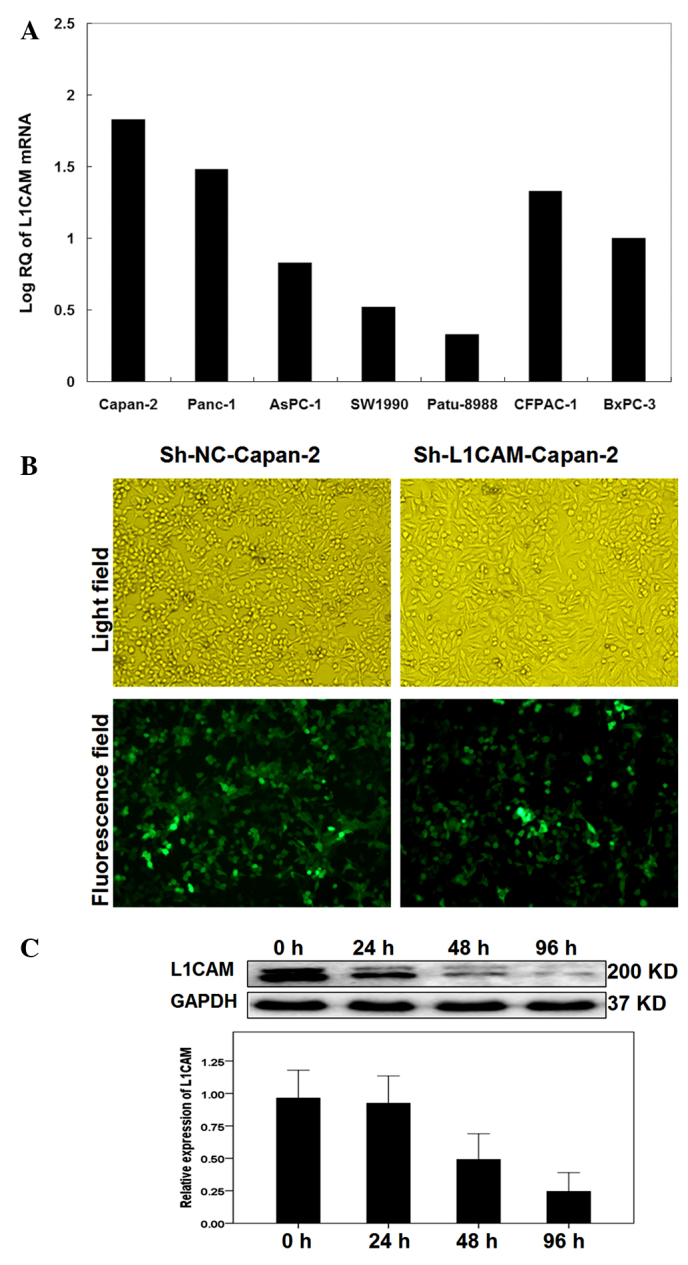
Lentivirus-mediated RNA interference decreased L1CAM expression in the pancreatic cell lines. (A) Polymerase chain reaction showed that human L1CAM mRNA was present in seven pancreatic cancer cell lines, Capan-2, PANC-1, AsPC-1, BxPC-3, SW-1990, Patu-8988 and CFPAC-1. L1CAM mRNA expression levels were highest in the Capan-2 cells and lowest in the Patu-8988 cells. (B) Light micrograph (top) and fluorescence micrograph (bottom) images (magnification, ×200). The transduction efficiency in the Capan-2 cells was estimated 96 h after infection and the intensity of fluorescence indicated a high transfection efficiency. (C) Western blot analysis of total cellular proteins extracted from Capan-2 cells at 0, 24, 48, 72 and 96 h after infection and targeted by antibodies against L1CAM, showed that the L1CAM protein levels decreased in a time-dependent manner. GAPDH served as the internal control. Data represents one of three independent experiments. L1CAM, L1 cell adhesion molecule; NC, negative control; Sh, short hairpin; RQ, relative quantity.

PCR analysis showed that L1CAM mRNA was present in the seven pancreatic cancer cell lines, Capan-2, PANC-1, AsPC-1, BxPC-3, SW-1990, Patu-8988 and CFPAC-1. The Capan-2 cells demonstrated the highest levels of L1CAM mRNA and the Patu-8988 cells displayed the lowest (Fig. 1A). Therefore, the Capan-2 cell line was used for the subsequent experiments.

### Infection efficiency in Capan-2 pancreatic cancer cells

The infection efficiency of Capan-2 cells with lentivirus-mediated sh-L1CAM was detected 96 h after infection by fluorescence microscopy. A high intensity of green fluorescence was observed, indicating a high infection efficiency (Fig. 1B). Western blot analysis indicated that the interfering efficiency was greatest in the Capan-2 cells 96 h after infection with ~75% reduction in L1CAM protein levels. Furthermore, the western blot analysis demonstrated that the L1CAM protein levels decreased in a time-dependent manner following infection (Fig. 1C).

### L1CAM silencing inhibits tumor cell proliferation and cell cycle entry in pancreatic cancer Capan-2 cells

**Figure 2:**
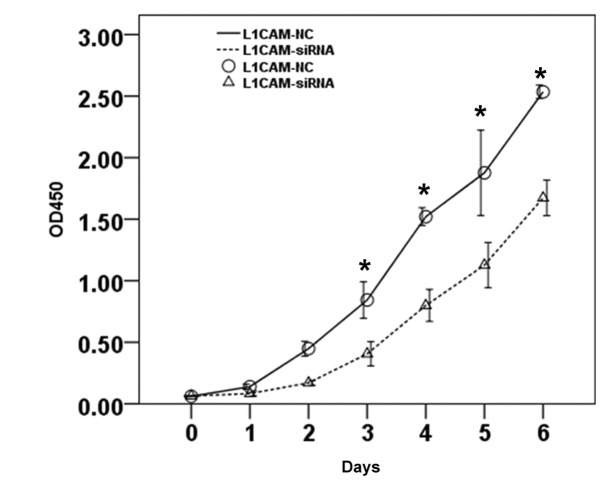
Effect of L1CAM silencing on Capan-2 cell proliferation. Cell viability was measured by cell counting kit-8 assays each day for six days (0–144 h). The results showed that LICAM silencing inhibited cell proliferation in the Capan-2 cells. Viability was expressed at an absorbance of 450 nm. L1CAM, L1 cell adhesion molecule; siRNA, small interfering RNA; OD, optical density.

**Figure 3:**
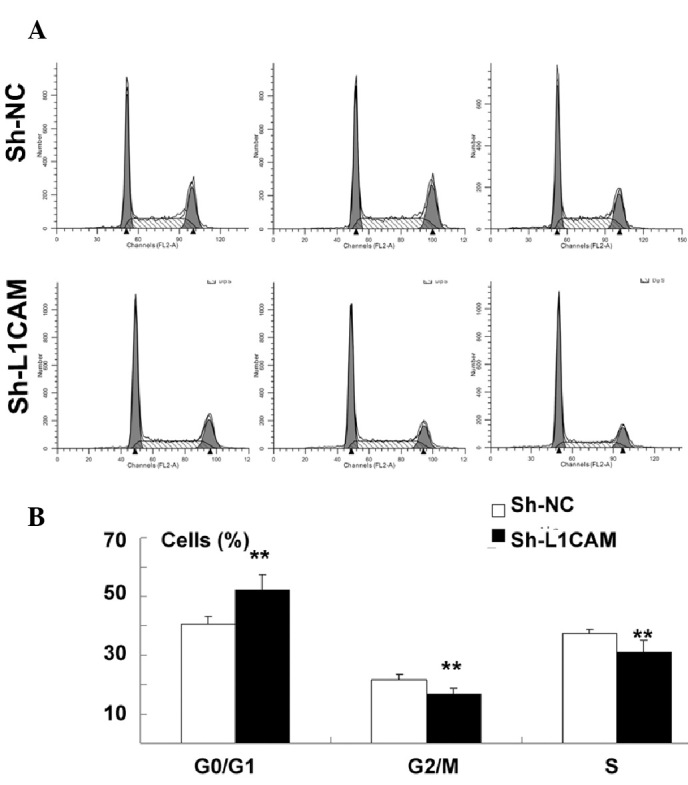
Effect of L1CAM silencing on Capan-2 cell cycle progression. (A) Flow cytometric histograms of the Capan-2 cells following silencing of L1CAM (Sh-L1CAM) in three parallel experiments. (B) Analysis of the cellular subpopulations at various phases of the cell cycle showed an increase in the percentage of cells at the G0/G1 phase, as well as a decrease in the percentage of cells at the G2/M and S phases, relative to the NC (Sh-NC). ^**^P<0.01, vs. Sh-NC. NC, negative control; L1CAM, L1 cell adhesion molecule; Sh, short hairpin.

The effect of L1CAM silencing on proliferation and cell cycle distribution in Capan-2 pancreatic cancer cells was determined by CCK-8 and flow cytometric assays, respectively. Proliferation was identified to be significantly inhibited by sh-L1CAM silencing when compared with the negative control (P<0.01; Fig. 2). In addition, there was a significant increase in the cell population at the G0/G1 phase, with a concurrent decrease in the cell population at the G2/M and S phases following L1CAM knockdown (P<0.01; Fig. 3A and B).

### L1CAM silencing inhibits tumor cell invasion but does not induce cell apoptosis in Capan-2 pancreatic cancer cells

**Figure 4:**
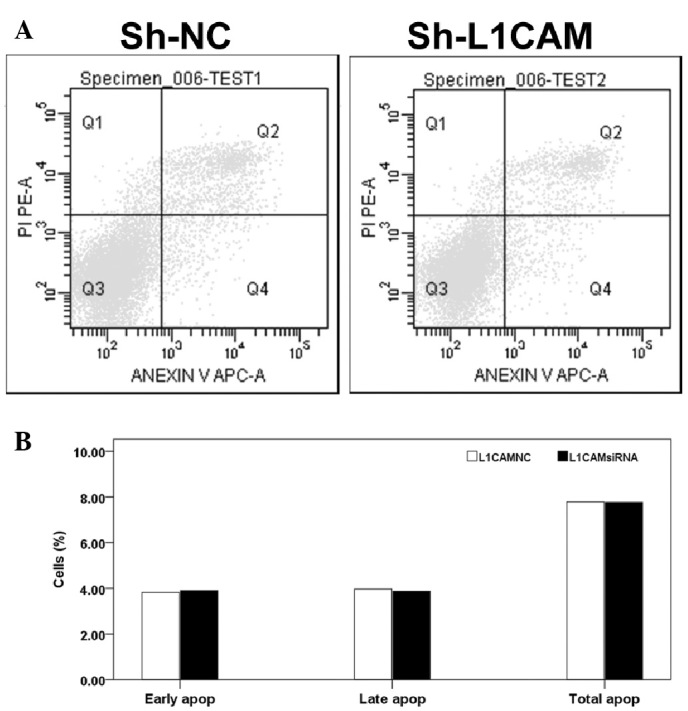
Effect of L1CAM silencing on apoptosis in Capan-2 cells. (A) Flow cytometric analysis demonstrating the effect of L1CAM silencing (Sh-L1CAM) on cell apoptosis. (B) Analysis of the cellular subpopulations during early apoptosis, late apoptosis and total apoptosis relative to the NC (Sh-NC) showed that the effect was not statistically significant (P>0.05). NC, negative control; L1CAM, L1 cell adhesion molecule; siRNA, small interfering RNA; Sh, short hairpin.

**Figure 5:**
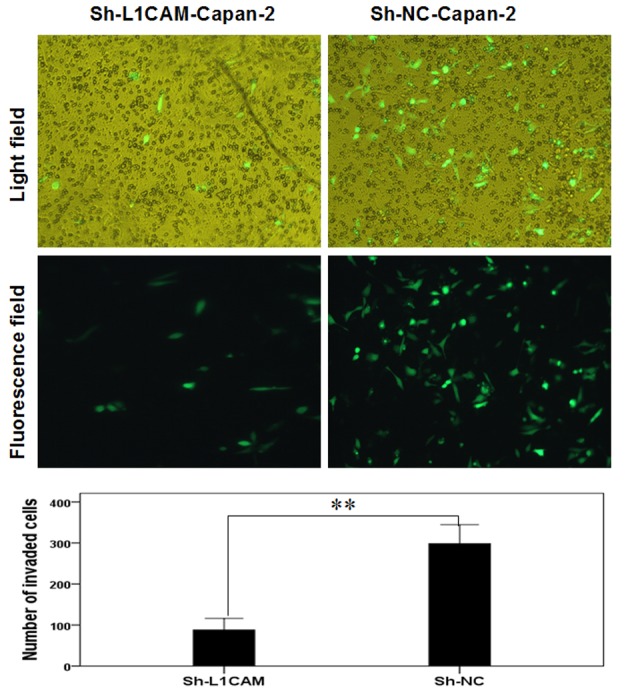
Effect of L1CAM silencing on the invasion of Capan-2 cells. The cell invasion assays in the matrigel-coated transwell chambers indicated that L1CAM silencing (Sh-L1CAM) significantly reduced cell invasion compared with the NC (Sh-NC). The invasive cells were counted in five random fields. ^**^P<0.01, vs. NC. L1CAM, L1 cell adhesion molecule; NC, negative control; Sh, short hairpin.

Next, the ability of L1CAM silencing to induce cell apoptosis was investigated. The results of Annexin V and PI flow cytometric analyses showed that lentivirus-mediated inhibition of L1CAM did not significantly increase apoptosis in the Capan-2 cells (P>0.05; Fig. 4A and B). By contrast, the number of invasive Capan-2 cells after sh-L1CAM silencing was identified to be significantly lower 96 h after infection compared with the negative control (P<0.01; Fig. 5).

### L1CAM silencing inhibits activation of extracellular signal-regulated kinase (ERK) in pancreatic cancer cells

**Figure 6:**
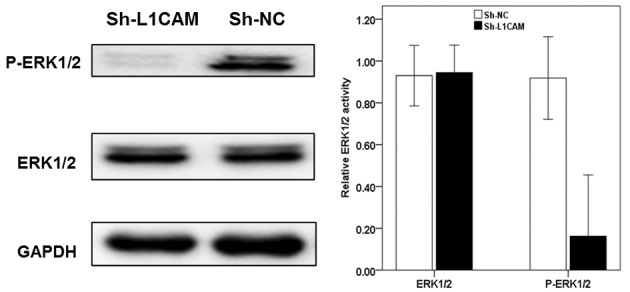
Effect of L1CAM silencing on ERK1/2 activation. L1CAM silencing (Sh-L1CAM) significantly reduced ERK1/2 activation in Capan-2 cells compared with the NC (Sh-NC). ^**^P<0.01, vs. Sh-NC. L1CAM, L1 cell adhesion molecule; ERK, extracellular signal-regulated kinase; NC, negative control; Sh, short hairpin.

p38 mitogen-activated protein kinase (MAPK) and ERK have been implicated in cancer metastasis signaling pathways and may be involved in regulating L1CAM activation (9,13). As shown in Fig. 6, it was identified that the downregulation of L1CAM inhibited the intrinsic activation of ERK1/2 in Capan-2 cells, indicating that L1CAM may be involved in cancer cell progression via the p38/ERK1/2 signaling pathway.

## Discussion

An understanding of the molecular mechanisms underlying cancer progression is essential for the development of optimal therapeutic modalities. Pancreatic cancer is a heterogeneous disease involving multiple cellular components and gene expression patterns. An increasing number of studies have identified the molecular markers that correlate with the development and progression of cancer.

RNA interference has been used as a therapeutic tool in cancer treatments (14), however, strategies to improve the methodology and efficiency of siRNA delivery are required. Although L1CAM mRNA was observed in the seven human pancreatic cancer cell lines that were investigated in the present study, the level was highest in the Capan-2 cells. Therefore, in order to assess the impact of L1CAM knockdown on the development of pancreatic cancer *in vitro*, Capan-2 cells were infected with lentivirus-mediated L1CAM-specific shRNA. Silencing of endogenous L1CAM significantly inhibited cell proliferation and invasion in the Capan-2 cells (P<0.01). In addition, L1CAM silencing induced cell cycle arrest at the G0/G1 phase, however, there was no significant effect observed on apoptosis. These results indicated that L1CAM is overexpressed in tumors and may be significant in cancer cell invasion.

Previous studies have indicated that L1CAM, a cell surface molecule, promotes tumor cell proliferation. Zecchini *et al* (15) showed that the upregulation of L1CAM in OVCAR3 ovarian cancer cells significantly enhanced cell proliferation in comparison to the parental cells. Conversely, the downregulation of L1CAM in IGROV1 ovarian cancer cells significantly inhibited cell proliferation (15). Furthermore, when tumor cells, including SKOV3 ovarian (16) and HCT116 colon cancer cells (17), were treated with an L1CAM monoclonal antibody, cell proliferation was reduced by 40–60% (18). *In vivo* studies demonstrated that cancer cells overexpressing L1CAM significantly promoted tumor formation, with tumor volumes that were 3–5 times greater than those with low levels of L1CAM expression (8). Kiefel *et al* (19) observed that the upregulation of L1CAM in pancreatic PT45-P1 cells promoted cell proliferation and xenograft growth.

L1CAM was initially hypothesized to be specific to the nervous system and has been implicated in numerous neurological disorders. However, L1CAM has recently been shown to be expressed in human tumors and correlate with tumor progression, poor prognosis and the advanced stages of cancer (9,10,20). Investigations in a variety of tumor types demonstrated that increased expression of L1CAM significantly increases the migration capacity of cancer cells *in vitro* (17,21–25). In addition, the upregulation of L1CAM was found to enhance cell invasion in the SW707 human colon cancer cell line (17). *In vivo* experiments have demonstrated that L1CAM significantly increases liver metastases in mice inoculated with LS174T colon cancer cells (26). In addition, multiple clinical pathology studies have indicated that L1CAM may promote cancer cell invasion and metastasis (10,27–31).

Although the mechanism by which L1CAM promotes proliferation and cell invasion in pancreatic ductal adenocarcinoma remains to be determined, studies have investigated the role of L1CAM-dependent activation in the MAPK-ERK signaling pathway. Schaefer *et al* (32) demonstrated that L1CAM induces ERK activity and ERK-regulated gene expression, which contributes to cell motility and invasion. Furthermore, L1CAM has been identified to interact with various components of the ERK pathway, including Src protein tyrosine kinases (33) and Ran-binding protein M (34), indicating that L1CAM may serve as an adaptor protein in L1CAM-induced ERK activation. Furthermore, L1CAM-signaling has been implicated in integrin-binding and the nuclear factor-κB pathways (35).

Epithelial-mesenchymal transition (EMT) is characterized by morphological and phenotypical alterations in cancer invasion and metastasis. Epithelial carcinoma cells acquire a motile phenotype via EMT, enabling the gain of metastatic potentials, whereas metastatic tumor cells often display a mesenchymal phenotype with a loss of epithelial markers, such as E-cadherin. A connection between L1CAM and EMT was initially identified by Shtutman *et al* (36) and it was observed that the expression of L1CAM in MCF7 mammary carcinoma cell lines disrupted E-cadherin-containing adheren junctions and increased the transcriptional activity of β-catenin. As L1CAM is a target gene of β-catenin (17), this event resulted in increased cell motility. In addition, treatment of pancreatic cancer cell lines with the EMT inducer, transforming growth factor (TGF)-β1, was found to upregulate L1CAM, leading to increased cell migration and invasion (35,37,38). These events have been implicated in the early stages of tumor development in pancreatic cancer (39). Geismann *et al* (39) observed that treatment with TGF-β1 caused H6c7 pancreatic ductal cells to acquire a spindle-shaped morphology, elevated cell migration potential and increased L1CAM expression. These effects may be abolished by the interference of TGF-β1 signaling or suppression of Slug (39). Conversely, L1CAM-mediated metastasis in colon cancer cells was shown to be independent of EMT induction and altered the expression of epithelial and mesenchymal marker proteins (40). Therefore, the impact of L1CAM on EMT requires further investigation.

In conclusion, the results of this study indicate that downregulation of L1CAM inhibits cell proliferation, induces cell cycle quiescence and reduces cell invasion in the Capan-2 pancreatic cell line. Thus, these effects may be associated with an observed decrease in p38/ERK expression. These findings indicate that L1CAM may be involved in metastatic potential and may, therefore, be a molecular target in anti-metastatic therapies for pancreatic cancer.
